# Isolation and Characterization of a Hybrid Respiratory Supercomplex Consisting of *Mycobacterium tuberculosis* Cytochrome *bcc* and *Mycobacterium smegmatis* Cytochrome *aa*_3_*

**DOI:** 10.1074/jbc.M114.624312

**Published:** 2015-04-10

**Authors:** Mi-Sun Kim, Jichan Jang, Nurlilah Binte AB Rahman, Kevin Pethe, Edward A. Berry, Li-Shar Huang

**Affiliations:** From the ‡ State University of New York Upstate Medical University, Syracuse, New York 13210,; the § Institut Pasteur Korea, Sampyeong-dong, Seongnam-si, Gyeonggi-do 463-400, Korea, and; the ¶ Lee Kong Chian School of Medicine and School of Biological Sciences, Nanyang Technological University, Singapore 639798, Singapore

**Keywords:** electron transfer complex, enzyme purification, Mycobacterium smegmatis, Mycobacterium tuberculosis, respiratory chain, Q203, cytochrome bcc, respiratory supercomplex

## Abstract

Recently, energy production pathways have been shown to be viable antitubercular drug targets to combat multidrug-resistant tuberculosis and eliminate pathogen in the dormant state. One family of drugs currently under development, the imidazo[1,2-*a*]pyridine derivatives, is believed to target the pathogen's homolog of the mitochondrial *bc*_1_ complex. This complex, denoted cytochrome *bcc*, is highly divergent from mitochondrial Complex III both in subunit structure and inhibitor sensitivity, making it a good target for drug development. There is no soluble cytochrome *c* in mycobacteria to transport electrons from the *bcc* complex to cytochrome oxidase. Instead, the *bcc* complex exists in a “supercomplex” with a cytochrome *aa*_3_-type cytochrome oxidase, presumably allowing direct electron transfer. We describe here purification and initial characterization of the mycobacterial cytochrome *bcc-aa*_3_ supercomplex using a strain of *M. smegmatis* that has been engineered to express the *M. tuberculosis* cytochrome *bcc*. The resulting hybrid supercomplex is stable during extraction and purification in the presence of dodecyl maltoside detergent. It is hoped that this purification procedure will potentiate functional studies of the complex as well as crystallographic studies of drug binding and provide structural insight into a third class of the *bc* complex superfamily.

## Teaser

**Background:** Mycobacteria have no soluble cytochrome *c*; the electron transfer chain involves a Complex III-IV “supercomplex.”

**Results:** Expression of the *M. tuberculosis* Complex III in *M. smegmatis* lacking native complex yields a functional hybrid supercomplex.

**Conclusion:** This supercomplex is a dimer of protomers containing two each of hemes A, B, and C.

**Significance:** This is the first purification of respiratory Complex III or IV from *Mycobacterium*.

## Introduction

*Mycobacterium tuberculosis* is the etiological agent of tuberculosis, an airborne disease responsible for 1.4 million deaths in 2013. Elimination of tuberculosis is complicated by the ability of the pathogen to survive for extended periods of time in a non-replicating state in granulomas and by the acquisition of mechanisms of resistance to all of the first and second line drugs used thus far. Development of new drugs targeting different pathways and effective against the latent form of the disease is thus a high priority (1). Recently, oxidative phosphorylation has been demonstrated as an effective target (2–4), and efforts are under way to increase understanding of the mycobacterial energy-producing pathways (2, 3, 5). A new drug that has recently been approved for clinical treatment, bedaquiline, targets the pathogen's ATP synthase enzyme. Although bedaquiline holds great promise for the treatment of multidrug-resistant and extensively drug-resistant tuberculosis, safety concerns still restrict the use of the drug to cases where other treatments have failed (6).

Another family of drug candidates, which has been shown to compromise the pathogen's energy production (7–9), is the imidazo[1,2-*a*]pyridine (IP)^8^ series. Two independent studies identified a single polymorphism in cytochrome *b* (the QcrB gene product) that confers high resistance to the IP series (8, 9). Another study, using a strain made hypersensitive by deletion of the cytochrome *bd* oxidase, identified seven more resistance-conferring mutations in cytochrome *b* (10).

The *QcrCAB* operon codes for the respiratory cytochrome *bcc* complex (11), which is a homologue of the mitochondrial cytochrome *bc*_1_ (Complex III) and the chloroplast *b*_6_*f* complexes. When the *M. tuberculosis* QcrB gene product sequence is aligned with *bc*_1_ and *b*_6_*f* sequences, all of the resistance-conferring mutations are near the Qp quinone-binding site, leading to the assumption that the IP drugs work by binding in the Qp site, thereby excluding the substrate (9, 10).

The original resistance mutation (8, 9) is T313A. This residue aligns with Ala-277 in human cytochrome *b*; thus, humans (and other vertebrates) have the resistance mutation in the normal state. Other factors are also required for sensitivity, because closely related actinobacteria, such as *Mycobacterium smegmatis*, have Thr in this position and yet are resistant. Thus, it is not surprising that preclinical studies of lead IP drug candidates have shown low toxicity in vertebrates (8, 9, 12). The leading drug candidate, Q203, combines this low toxicity with high activity against multidrug-resistant and extensively drug-resistant clinical isolates and high potency at low dose in a mouse model of tuberculosis (8, 9, 12).

**FIGURE 1.**
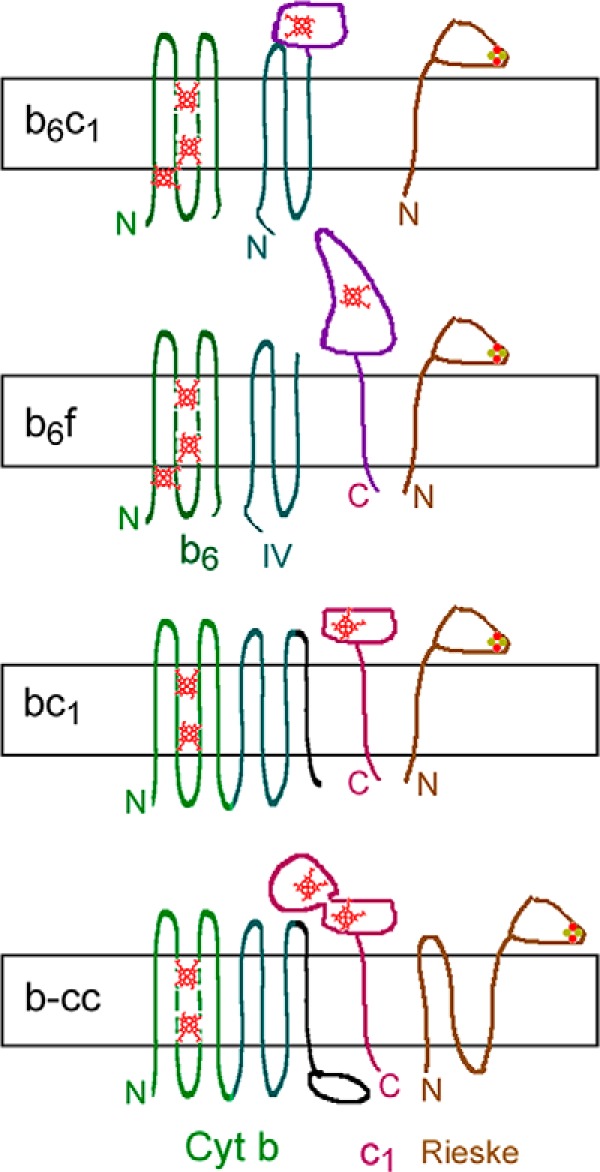
**Four divisions of *bc* complexes.** *N* and *C*, N and C termini; *small red figures*, hemes. The *rectangular slab* represents the energy-transducing membrane, with P side *above* and N side *below*. The mycobacterial *bcc* complex cytochrome *b* has sequences corresponding to *b*_6_ and SU_IV_ in a single polypeptide like mitochondrial cyt *b* but with an extension on the C terminus. The c-type cytochrome has two hemes and is the only *c*-type cytochrome in the genome. The iron-sulfur protein apparently has three transmembrane helices.

Both mitochondrial cytochrome *bc*_1_ (Complex III) and mycobacterial cytochrome *bcc* belong to an evolutionarily related superfamily of “cytochrome *bc*” complexes catalyzing electron transfer from a hydrophobic quinol to a proteinaceous acceptor (13). The members have three redox cofactor-bearing proteins: cytochrome *b*, a *c*-type cytochrome, and an iron-sulfur protein of the Rieske type. The cytochrome *b* and iron-sulfur protein sequences are evolutionarily related,, whereas the *c*-type cytochromes appear to be unrelated. The superfamily has four major subdivisions differentiated (among other things) by whether the cytochrome *b* sequences are present on one polypeptide or split into two and the type of *c* cytochrome (Fig. 1). The most familiar examples are the mitochondrial or proteobacterial cytochrome *bc*_1_ complexes and chloroplast or cyanobacterial *b*_6_*f* complexes. For the *bc*_1_ complexes, the minimal unit consists of cytochrome *b* with eight transmembrane helices (TMHs), cytochrome *c*_1_ with a single C-terminal TMH, and a Rieske-type high potential Fe_2_S_2_ iron-sulfur complex with a single N-terminal TMH.

The *b*_6_*f* complexes have cytochrome *b* sequences split over two subunits. Cytochrome *b*_6_ has sequences homologous to the first four TMHs of cyt *b*, which form a four-helix bundle enclosing the two hemes (*green* in Fig. 1). Subunit 4 (SU_IV_) has sequences corresponding to TMHs 5–7 of cyt *b* (*blue-green*), including the highly conserved PEWY sequence that lines one side of the quinol-oxidizing Qp site. They have an unrelated *c* cytochrome (cytochrome *f*) and a Rieske protein.

The *b*_6_*c*_1_ complexes of firmicutes (Gram-positive organisms with low GC content) typified by *Geobacillus stearothermophilus* (formerly *Bacillus stearothermophilus*) have a short cyt *b* subunit like *b*_6_ (14). The three transmembrane helices homologous to SU_IV_ are fused to a cytochrome *c*, which is not closely related to either mitochondrial cyt *c*_1_ or cytochrome *f*.

The cytochrome bcc complexes of GC-rich Gram-positive bacteria (actinobacteria) such as *Mycobacterium*, *Corynebacterium*, and *Rhodococcus* have a long cyt *b*, containing sequences aligning with *b*_6_ and SU_IV_, and a C-terminal extension of about 115 residues (11, 15). The Rieske protein is similar in sequence to the recently solved Rieske protein from *Thermus thermophilus* but has a long N-terminal extension with apparently three transmembrane helices. There is a diheme *c*-type cytochrome (cyt *cc*), with a C-terminal TMH (11, 15). It has been suggested that one cytochrome *c* domain is the donor for cytochrome oxidase, and the other one is the acceptor from the Rieske protein (11, 15). There is no soluble cytochrome *c* or any other *c*-type cytochrome at all in the genome. The two complexes form a supercomplex that facilitates electron transfer from menaquinol to oxygen (15–17).

QcrB is predicted to be essential based on transposon mutagenesis experiments (18, 19). Furthermore, the *bcc-aa*_3_ pathway could not be deleted by homologous recombination in *M. tuberculosis* (20), reinforcing the notion that it is vital for *M. tuberculosis*. Assuming that the IPA series target the *bcc* complex, such observations could explain the effectiveness of the series against *M. tuberculosis in vitro* and *in vivo* (8, 9, 12). Interestingly, disruption of the *bcc-aa_3_* pathway is possible in *M. smegmatis* but leads to a profound growth impairment (20).

The essentiality of the cytochrome bcc in *M. tuberculosis*, together with the large differences between this menaquinol-oxidizing enzyme of the pathogen and the ubiquinol-oxidizing human mitochondrial *bc*_1_ complex, make it a good target for therapeutic intervention to control or eliminate tuberculosis.

The genes for the three cytochrome *bcc* subunits *qcrC* (cyt *cc*), *qcrA* (iron-sulfur protein), and *qcrB* (cyt *b*) are organized into the *qcr* (quinol cytochrome *c* reductase) operon. The cyt *aa*_3_ cytochrome oxidase subunits are encoded by the genes *ctaC* (Cox2), *ctaD* (Cox1), and *ctaE* (Cox3).

We report here the purification and initial characterization of a stable supercomplex consisting of the cytochrome *bcc* of *M. tuberculosis* and cytochrome *aa*_3_ of *M. smegmatis*, using untagged proteins and methods suitable for purification on a scale large enough to support crystallographic studies. We believe that this is the first reported isolation an untagged Complex III or IV from actinobacteria and the first tagless isolation of a stable Complex III-IV supercomplex from any organism.

## Experimental Procedures

### 

#### 

##### Bacterial Strains

*M. smegmatis* Δ*qcrCAB* (20) (kindly provided by Valerie Mizrahi and Bavesh Kana) was transformed with a replicative plasmid, pMV262, harboring the full *qcrCAB* operon from *M. tuberculosis* under the control of the hsp60 promoter. The 3775-bp fragment encoding the complete *qcrCAB* was amplified by polymerase chain reaction (PCR) from *M. tuberculosis* genomic DNA using the oligonucleotides with the following sequences: 5′-TCGAAGCTTATCGATATGACGAAACTGGGGTTCACCC-3′ and 5′-CGTACGCTAGTTAACCTAGTGCTCGCCGTCTGGCG-3′ that contain a ClaI and an HpaI restriction site (underlined), respectively. The PCR product was digested with ClaI and HpaI and then inserted into the ClaI and HpaI sites of the *Escherichia coli*-mycobacteria shuttle expression vector pMV262 to yield pMV262-*qcrCAB*. The pMV262-*qcrCAB* was finally transformed by electroporation into *M. smegmatis* Δ*qcrCAB*::hyg. The recombinant strain was maintained on kanamycin (25 μg/ml) and hygromycin (50 μg/ml). Antibiotics were omitted from cultures intended for protein purification.

##### Mycobacterial Culture

Cells were grown in Bacto brand soy tryptic broth supplemented with Tween 80 (0.5 ml/liter), at 30 °C with shaking to maintain oxygenation. Cells were grown from frozen stock in three stages (3 ml, 100 ml, and 6 1L). After the *A*_600_ reached ∼1.5, the bacteria were harvested by centrifugation and resuspended in 50 mm KP_i_, 0.5 mm EDTA, and PMSF and lysed either by two passages through a French pressure cell at 16,000 p.s.i. or by treatment with lysozyme and sonication. After a low speed centrifugation (1000 rpm for 5 min in the JA14 rotor (150 × *g* at *r*_max_) to remove intact cells, the light yellow membranes were pelleted by centrifuging at 50,000 rpm for 4 h using a Ti70 rotor (257,000 × *g*). The membranes were resuspended to a concentration of 35 g/liter protein in 50 mm KP_i_, 0.5 mm EDTA, and 0.1 mm freshly added PMSF and frozen at −80 °C until use.

##### Assays

Columns were monitored during the run by absorbance at 280 nm. Elution profiles of cytochromes were determined by scanning spectra from 340 to 470 (in the fraction collector tubes) and either fitting the spectra with a standard Soret peak spectrum or measuring the absorbance difference, 415 − 371 nm. To identify the cytochromes present, portions of selected fractions were diluted and analyzed by recording spectra from 250 to 650 nm before and after reducing with dithionite. Column fractions were analyzed by SDS-gel electrophoresis, scanning the gels for fluorescent cofactors with a Typhoon 9410 imager (GE Healthcare) as described (21) to locate covalent flavoproteins; heme (peroxidase) staining (22) to locate cytochrome *cc* with its covalently bound hemes; and Coomassie Brilliant Blue R for protein bands. Fractions containing supercomplex were identified by the presence of cyt *aa*_3_ in the spectrum and/or the presence of cytochrome *cc* from spectra of ascorbate-reduced fractions or heme-stained gels. Heme was quantified in the final product by making the pyridine hemochrome as described before (23), except that K_3_Fe(CN)_6_ was omitted, and absolute reduced spectra rather than reduced-minus-oxidized spectra were used for calculating heme content.

##### Purification

Membranes were thawed and diluted to a final concentration of 20 g/liter protein in 50 mm KP_i_, pH 7.4, 100 mm NaCl, and 10 g/liter *n*-dodecyl β-d-maltoside (DDM). The mixture was stirred magnetically at 4 °C for 30 min and centrifuged at 18,000 rpm for 20 min using a JA20 rotor (39,000 × *g*). The yellow supernatants were decanted and pooled, whereas the large white pellets were discarded.

The supernatant (up to 70 ml) was diluted with 0.25 volume of cold water and loaded onto a column of DEAE-Sepharose CL6B (100 ml; 2.6 × 30 cm) equilibrated with 50 mm KP_i_, 100 mm NaCl, 0.5 mm EDTA, and 0.1 g/liter DDM. (In some cases, 20 mm KMOPS replaced the 50 mm KP_i_). After loading, the column was washed with 100 ml of the equilibration buffer and eluted with a linear gradient from that buffer to a high salt buffer containing 50 mm KP_i_, 500 mm NaCl, 0.5 mm EDTA, and 0.1 g/liter DDM in 200 ml. This was followed by 100 ml of the high salt buffer.

Fractions containing cytochrome *bcc-aa*_3_ were pooled and concentrated by ultrafiltration (100 kDa cut-off membrane) before layering on top of glycerol density step gradients in tubes compatible with a SW-32.Ti rotor. The gradient was made by layering 10, 14, and 10 ml of 60, 40, and 20% (w/v) glycerol, 20 mm KMOPS, 100 mm NaCl, and 0.5 mm EDTA. The gradients were then centrifuged at 30,000 rpm for 72 h and fractionated by pumping out from the bottom, through a stainless steel cannula inserted down from the top of the tube, into a fraction collector. The gradients were fractionated one after another into the same set of tubes, which were then analyzed by Soret spectra, full dithionite-reduced spectra of selected fractions, and gel electrophoresis.

Fractions containing cytochrome *bcc-aa*_3_ were pooled and loaded without dilution onto a 7 × 1.5-cm Q-Sepharose column equilibrated with the same equilibration buffer as for the first (DEAE) column and eluted similarly but with 30, 100, and 45 ml for wash, gradient, and high salt wash.

Fractions containing the cytochrome *bcc-aa*_3_ were pooled, concentrated to 3 ml by ultrafiltration, and chromatographed on a 1.5 × 113-cm (200 ml) column of Sepharose CL-6B equilibrated with 20 mm KMOPS, 100 mm NaCl, 0.5 mm EDTA, and 0.1 g/liter DDM. Fractions containing cyt *bcc-aa*_3_ were pooled and concentrated and, in some cases, further purified on a second glycerol density gradient.

## Results and Discussion

### Expression of the M. tuberculosis qcrCAB Operon in M. smegmatis ΔqcrCAB Strain Restores Robust Growth

**FIGURE 2.**
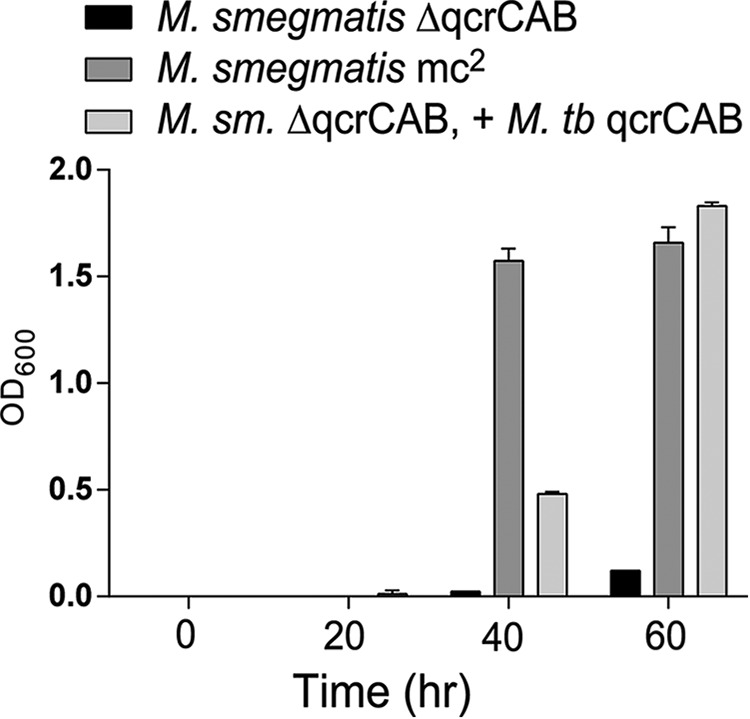
**Expressing *M. tuberculosi*s *qcrCAB* (cyt *bcc*) overcomes to a large extent the growth defect of *M. smegmatis* Δ*qcrCAB*.** Rich growth medium (7H9 Middlebrook broth plus 0.2% glycerol, 0.05% Tween 80, supplemented with 10% ADS (0.5% bovine albumin fraction V, 0.2% glucose, 140 mm NaCl)) was inoculated with the indicated strain at time 0, and *A*_600_ was read at the indicated times. The *error bars* show S.D. from triplicate cultures of each strain. The strains are described under “Experimental Procedures.”

The *M. tuberculosis bcc* complex (QcrCAB) was overexpressed in the strain *M. smegmatis* Δ*qcrCAB*::hyg. The *M. smegmatis* strain deficient for the expression of the *bcc* complex was previously shown to be viable but severely attenuated for growth (20). In Fig. 2, we show that the expression of the *M. tuberculosis bcc* complex restored the growth of the *M. smegmatis* Δ*qcrCAB* to a large extent. This result shows that the *M. tuberculosis bcc* complex is inserted into the membranes and can functionally complement the absence of the native *M. smegmatis* complex.

### Electron Transport Properties of the Membranes Prepared from M. smegmatis

**FIGURE 3.**
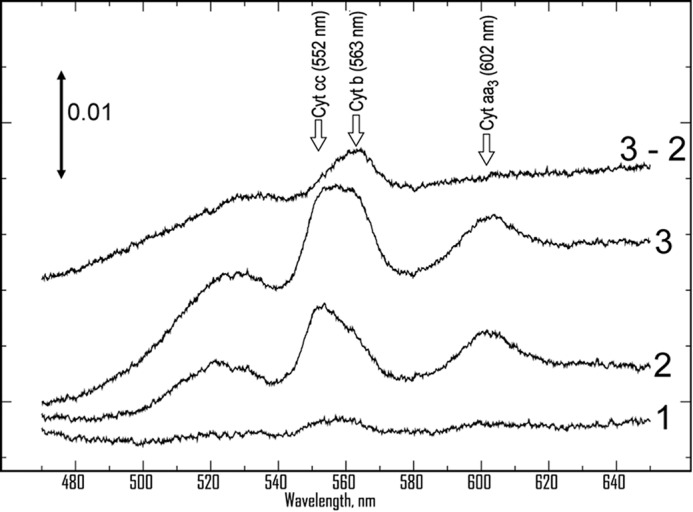
**Absorbance spectra of cytochromes in the membranes of *M. smegmatis*.** Membranes were diluted (to 0.6 g/liter protein in 50 mm KP_i_, 0.5 mm EDTA, pH 7.5), and a baseline was recorded, which has been subtracted from the subsequent spectra. NADH was added, and spectra were repeatedly scanned until the sample became anoxic, as indicated by increased reduction of cytochromes (*trace 2*), as compared with *trace 1* taken while O_2_ was present. Finally, sodium dithionite was added (*trace 3*), leading to additional reduction of *b*-type cytochrome(s), as indicated by the difference spectrum (*trace 3* − *2*). The spectra have been displaced vertically by arbitrary amounts to avoid overlap.

Membranes from *M. smegmatis* or *M. smegmatis* Δ*qcrCAB*_pMV262-MtbQcrCAB catalyze rapid oxidation of NADH. Fig. 3 shows spectral changes of membranes suspended in aerobic buffer after adding NADH. Only a slight reduction of cytochromes *aa*_3_ and *cc* is seen (Fig. 3, *trace 1*) until the medium becomes anoxic. At that point, *c*- and *a*-type cytochrome(s) are reduced, as indicated by peaks at 552 and 600 nm (*trace 2*). The *b* cytochromes are partly reduced (shoulder at 562 nm). Upon adding dithionite (*trace 3*), *b* cytochromes become further reduced, giving a flat-topped peak extending from 552 to 563 nm. The spectrum of the additional cytochromes reduced by dithionite has its peak at 563 nm (*trace 3* − *2*).

### Purification

We were able to purify the intact *bcc-aa*_3_ complex from *M. smegmatis* Δ*qcrCAB* expressing the *M. tuberculosis qcrCAB* operon by a procedure involving membrane extraction with DDM, two ion exchange chromatographies, density gradient centrifugation, and size exclusion chromatography. Extraction of membranes with 0.5 g of DDM/g of protein solubilized essentially all of the cytochromes, as determined by pyridine hemochrome analysis of the supernatant and pellet (not shown). From this extract, the cyt *bcc-aa*_3_ supercomplex was purified as described under “Experimental Procedures.”

#### 

##### Anion Exchange Chromatography Begins the Separation and Reveals the Different Spectral Signatures of Different Cytochrome Complexes

**FIGURE 4.**
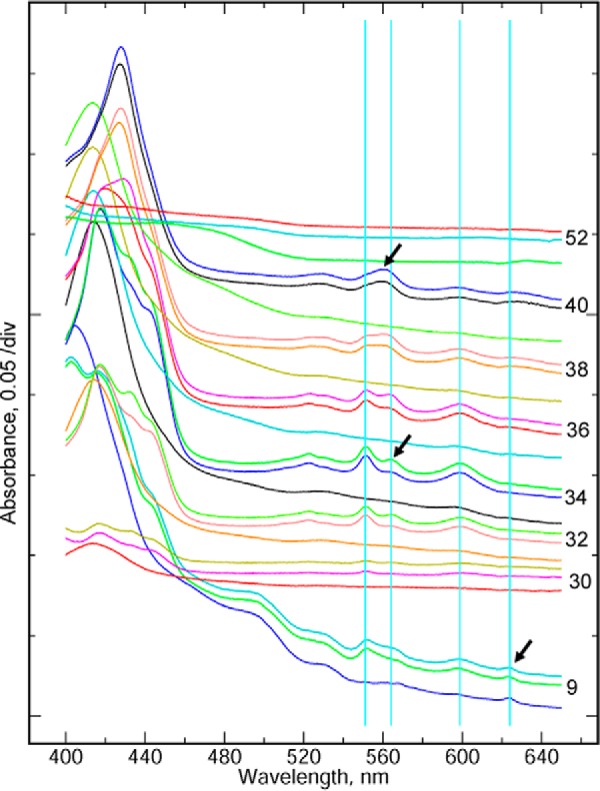
**Fractionation of cytochromes in the detergent extract by ion exchange chromatography.** Fractions from the column were analyzed by UV-visible spectroscopy. Three spectra are shown for each fraction: untreated and two consecutive scans after adding dithionite. Each triplet is *labeled* with the *fraction number*. For clarity, the spectra are offset by an arbitrary amount to order them from bottom to top while avoiding overlap in the range 480–650 nm. *Light blue vertical lines* indicate the wavelengths corresponding to absorbance peaks of the cytochrome *cc* (552 nm), cytochrome *b* (563 nm), and cytochrome *aa*_3_ (600 nm) of the supercomplex and cytochrome *d* (623 nm) of the cyt *bd* menaquinol oxidase.

First, a large DEAE-Sepharose column was used to obtain a crude fractionation. Spectra of selected fractions from this column are shown in Fig. 4. In this case, the supernatant was not diluted before applying, and the flow-through fractions contained some *c*- and *a*-type cytochromes. Even with dilution before loading, a small amount of *c*- and *a*-type cytochromes did not bind to the column, which may represent cytochromes not assembled into the supercomplex.

More significantly, the flow-through fractions have a peak at 623 nm. This is presumed to be cytochrome *d*, although 628–630 nm is more typical for that cytochrome (24). If so, then the peaks at ∼600 and ∼560 nm probably contain contributions from cyt *b*_595_ and *b*_558_ of the cyt *bd* oxidase. Cyt *d* is not seen in any other fractions, implying that the *bd* oxidase does not adsorb to the DEAE-Sepharose under these conditions. The 623 nm peak is already visible before adding dithionite. A two subunit cytochrome *bd* menaquinol oxidase has been purified from *Corynebacterium glutamicum* (25). The flow-through also contained a yellow, organic-soluble pigment, perhaps a carotenoid, with peaks at 475 and 445 nm and perhaps a third peak around ∼420 nm, which is obscured in the native spectra by the Soret peaks of cytochromes.

In the fractions eluted by the salt gradient, the flat-topped peak in the region of cyt *b* and *c* α peaks that was seen in the membranes splits into two spectral signatures. The fractions eluting early in the gradient (Fig. 4, fractions 32, 34, and 36) have the typical *bcc-aa*_3_ spectrum with well separated α peaks for cyt *b* (562 nm) and *cc* (552 nm). Cyt *b* is reduced slowly (and incompletely on this time scale), as indicated by the further reduction of cyt *b* in the second scan after adding dithionite. Later fractions (fractions 38 and 40) show a cyt *b*_557_ filling the trough between the 552 and 562 peaks, resulting in a single maximum, dominating the α peak in fraction 40. Fluorescence scans of SDS gels (not shown) reveal a high molecular weight flavoprotein in these fractions, suggesting that *b*_557_ belongs to the superfamily of succinate:quinone oxidoreductases (26).

Ion exchange chromatography did not completely separate cyt *b*_557_ from the supercomplex, but this can be achieved by density gradient centrifugation. In order to obtain a good yield, all of the fractions, eluted by the gradient, that contained cyt *aa*_3_ (as seen by the −600 nm spectral peak) were pooled for the next step, including some that contained significant amounts of the cyt *b*_557_ and flavoprotein.

##### Density Gradient Centrifugation Separates Supercomplex from Cyt b_557_

**FIGURE 5.**
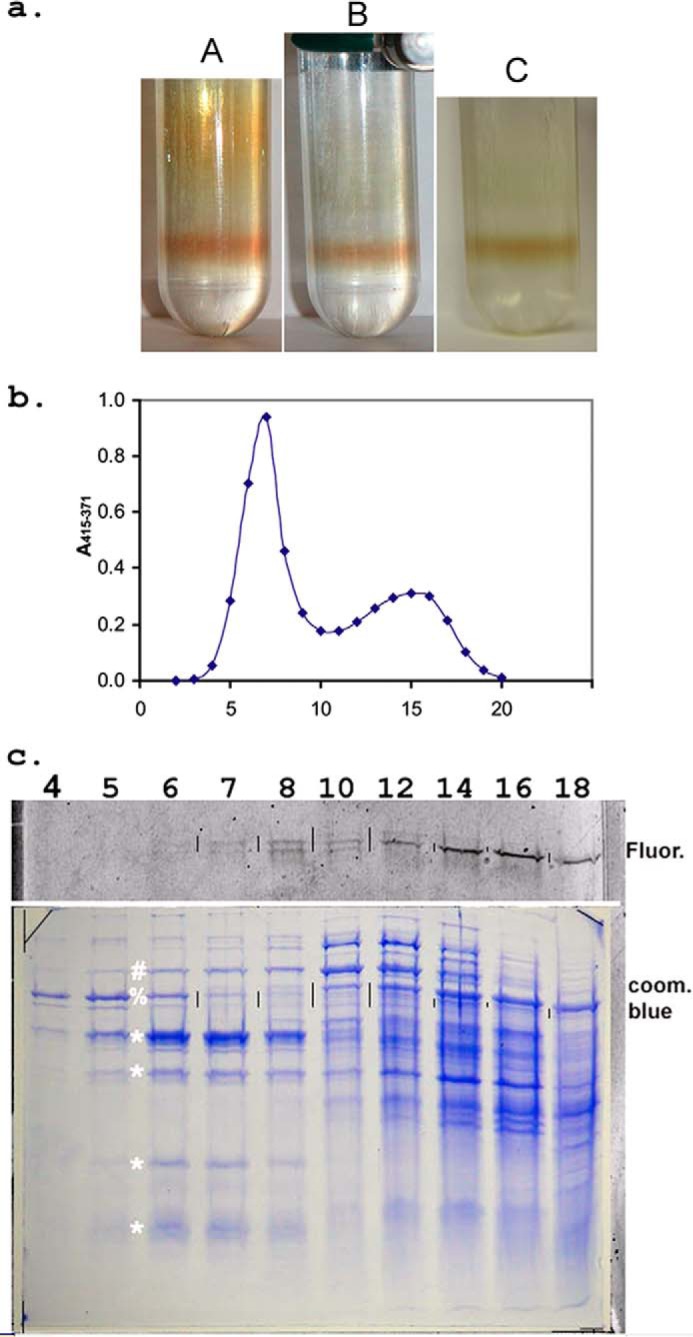
**Density gradient purification of cyt *bcc-aa*_3_.** Samples were separated by density gradient sedimentation as described under “Experimental Procedures.” *a*, supercomplex forms a sharp, visible band. In *A*, material from the initial ion exchange column was applied, demonstrating separation of slower moving *colored* contaminants. *B* and *C* both show the another sample with different illumination. In this case, the density gradient was applied as a final polishing step to nearly pure material. *A* and *B* are photographed with flash which gives a *redder* appearance, whereas C was photographed with fluorescent light and represents the *greenish-brown* appearance when viewed by eye under fluorescent lights. *b*, density gradient profile of cytochromes in pooled fractions from the initial DEAE-Sepharose column. The density gradient of Fig. 1*A* was fractionated by inserting a cannula to the bottom of the tube (from the top) and pumping to a fraction collector. The fraction collector was started at the same time as the pump, and fluid reached the fraction collector just before the start of fraction 2. The 20 fractions collected represent the bottom two-thirds of the gradient, with fraction 2 being the bottom. *c*, SDS-gel analysis of fractions from density gradient. The *bottom panel* shows the Coomassie-stained gel, and the *strip above* it is from a fluorescence scan of the gel. Cyt *bcc-aa*_3_ elutes primarily in fractions 6, 7, and 8. The four bands marked by *asterisks* are present in the purified complex, as is a sharp but weak band just below the largest of these. The putative identity of the bands will be provided later. The band indicated by # in *lanes 6–8* may be the heme-staining subcomplex described later and/or overlap from a slower sedimenting band of the same gel mobility seen in fractions 10–14. The 65 kDa band (indicated by %) sediments faster than *bcc-aa*_3_ but overlaps somewhat. It can be cleanly separated by ion exchange in the next step. The *vertical lines* between some of the lanes serve to register the fluorescent scan with the Coomassie-stained gel and indicate the position of fluorescent proteins on the latter.

Fig. 5 shows results from density gradient centrifugation. Photographs in Fig. 5*a* show a sharp colored band formed near the bottom of the tube, in what was originally the 60% glycerol layer. It is well separated from other cytochrome complexes due to its high molecular weight. This is not equilibrium centrifugation, and the supercomplex would eventually pellet if the run were long enough. However, the rate is greatly decreased upon entering the 60% glycerol, resulting in sharp focusing of the band, so the product can be recovered in a small volume. The perceived color depends on the illumination. When the relatively impure pooled fractions from the first ion exchange column were applied (tube A), the bulk of other cytochromes was well separated. Elution profile monitored by the Soret peak height (Fig. 5*b*) shows near-baseline separation between supercomplex peaking in fraction 7 and other cytochrome(s) peaking around 15. If the separation here is not good enough, a second density gradient centrifugation is performed as a final purification step.

Fig. 5*c* shows SDS-gel analysis of the fractions from the density gradient, probed by Coomassie stain and flavin fluorescence. Four or five bands (Fig. 5*c*, *) sedimenting in parallel, which peak in fractions 6 and 7, will be seen later to be characteristic of the supercomplex. A band at about 65 kDa (Fig. 5*c*, %) that sediments faster (peaking in fraction 5) but overlaps with the supercomplex will be well separated by the Q-Sepharose column in the next step.

The *top panel* in Fig. 5*c* shows fluorescence in the range of succinate:quinone oxidoreductase flavoproteins. There appear to be at least three fluorescent proteins based on gel mobility. The uppermost faint band is only visible in fractions 10–12. The middle band has a weak peak in fraction 8, after the peak of supercomplex but overlapping, and then a strong peak in fraction 14, well after supercomplex. The lower band peaks in fraction 8, in parallel with the first peak of the middle band. Given the sensitivity of the fluorescence detection, the flavoprotein overlapping supercomplex is present in a relatively insignificant amount already at this stage and would not be visible in the Coomassie-stained gel. On the other hand, the strong fluorescent band in fractions 14 and 16 does seem to correspond to a faint band in the Coomassie-stained gel, as indicated by *vertical black lines* placed between the lanes at this level in both fluorescent and stained gels for alignment. Given that the second heme peak is also in fractions 14–16 (Fig. 5*b*), this fluorescent band may be the flavoprotein of the putative cytochrome *b*_557_/succinate:quinone oxidoreductase, which was overlapping with the supercomplex on the ion exchange column of Fig. 4. Expression and function of two SQOR protein complexes of *M. smegmatis*, denoted SDH1 and SDH2, have been investigated (27).

##### A Second Anion Exchange Chromatography on Q-Sepharose

**FIGURE 6.**
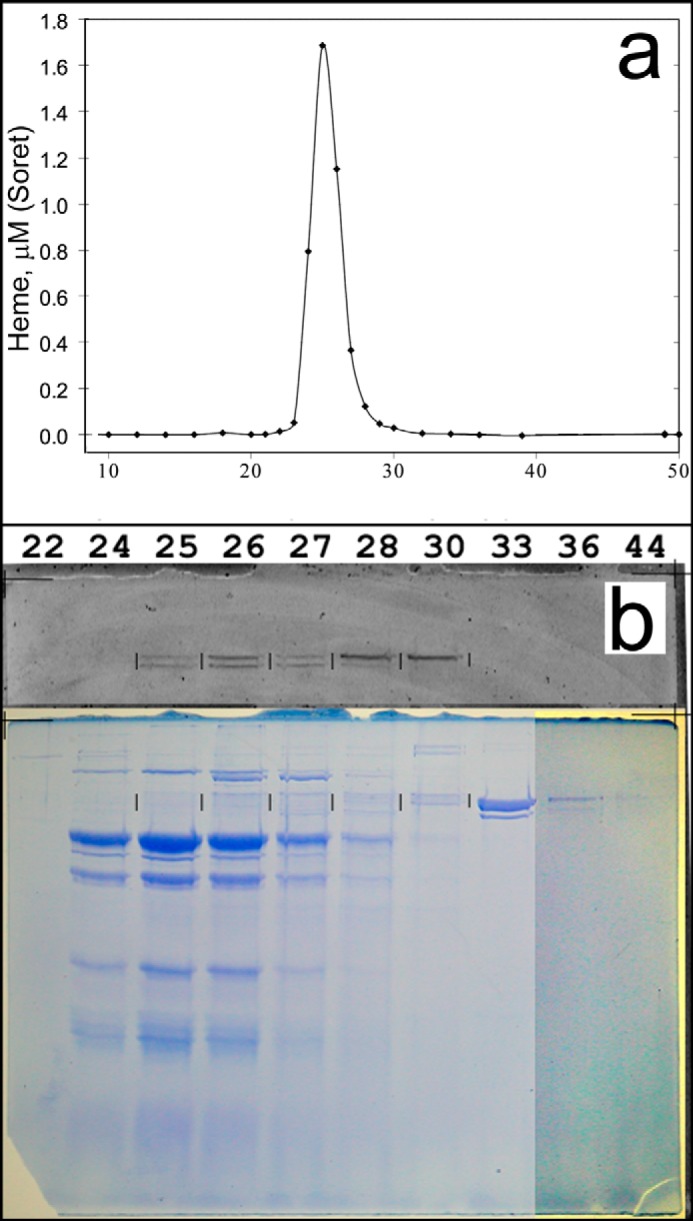
**Further purification by anion exchange chromatography on Q-Sepharose.** *a*, elution profile of heme measured by fitting the Soret peak to a 1 μm standard spectrum. *b*, SDS-PAGE analysis of fractions during gradient elution. The *top portion* shows the fluorescence scan, and fraction numbers are indicated at the *top*. Supercomplex elutes in fractions 24–27. Note that the 65 kDa band in fraction 33 is the material sedimenting faster than supercomplex in the density gradient (Fig. 4) and is well separated in this step. Contrast was enhanced for the Coomassie-stained gel in lanes 36 and 44 to show weak bands. Fraction size was 3 ml; the salt gradient eluted in approximately fractions 15–48.

Due to the relatively low ionic strength of the buffer used for the density gradient, pooled fractions can be bound to Q-Sepharose resin without further dilution. A 12-ml Q-Sepharose column was used, with the same washing and eluting buffer as for the first DEAE-Sepharose column, but with volumes of 30 ml for washing and 100 ml for the linear gradient. SDS-gel analysis of fractions around the supercomplex peak is shown in Fig. 6. Supercomplex elutes in fractions 24–27. As mentioned above, the 65 kDa band elutes well after the supercomplex, peaking in fraction 33. The main flavoprotein peak elutes after the supercomplex, but two weaker fluorescent bands elute around fraction 26, overlapping supercomplex. Again, given the sensitivity of the fluorescent detection, this represents a very slight contamination by these flavoproteins, barely detectable by Coomassie staining at the level of the *vertical marks*.

##### Size Fractionation by Gel Filtration

**FIGURE 7.**
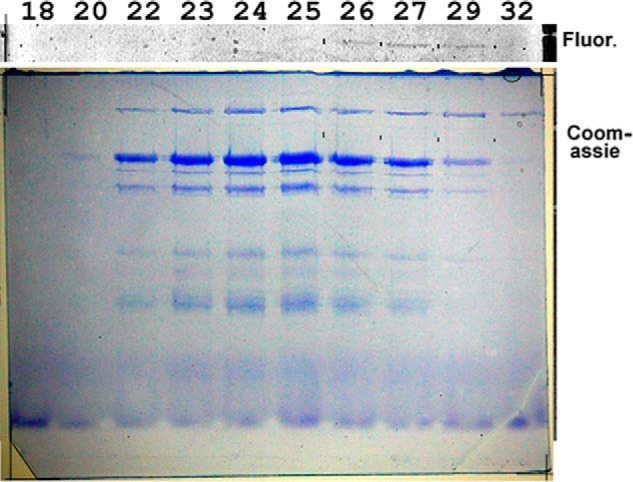
**Sepharose CL-6B size exclusion chromatography.** Samples from fractions across the elution peak were denatured and separated by SDS gel electrophoresis. The gel was first scanned for flavin fluorescence (top) and then stained with Coomassie Brilliant Blue R. *Fine black marks between* the *lanes* on both indicate the locations of fluorescent bands due to flavoprotein.

Pooled fractions from the Q-Sepharose column were chromatographed on Sepharose CL-6B. Fig. 7 shows SDS gel analysis of fractions eluting near supercomplex. Fluorescence due to flavoprotein is barely detectable now, and the flavoprotein elutes slightly after, but still overlapping with the supercomplex. These bands are not detected by Coomassie staining. The Coomassie-stainable bands elute in constant proportion and presumably belong to the supercomplex, except for the highest, above the flavoprotein. This band appears together with the supercomplex and peaks in fractions 24 and 25, but continues to elute as late as fraction 32. The molecular weight is too high for any known subunits of the supercomplex, but as discussed below, an incompletely dissociated complex containing cytochrome *cc* is often seen near this molecular weight. This subcomplex may be responsible for that portion of the high molecular weight band in Fig. 7 peaking in fractions 24 and 25 with supercomplex, but in that case, an impurity of the same gel mobility must be overlapping in order to account for this band extending to fraction 32, where the other supercomplex bands are undetectable.

##### Summary of the Purification

**FIGURE 8.**
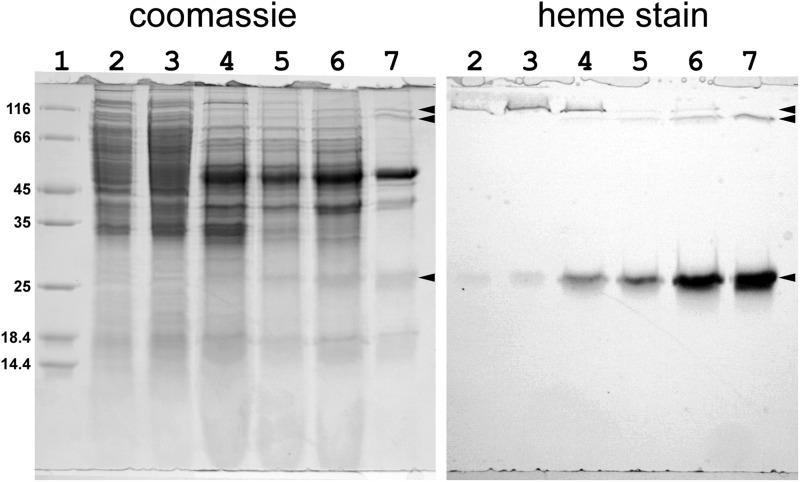
**SDS-PAGE analysis at different stages of purification.** *Lane 1*, molecular weight standards; *lane 2*, membranes; *lane 3*, detergent extract; *lane 4*, pooled fractions from DE-Sepharose column; *lane 5*, after Sepharose CL6b; *lane 6*, Q-Sepharose; *lane 7*, glycerol density gradient. *Left*, stained with Coomassie Brilliant Blue R for protein. *Right*, heme stain. *Arrows* on the *right* of both pictures indicate the positions of three heme-staining bands. The two high molecular weight heme-staining bands are not seen if the samples are heated near boiling to denature. The highest, present especially in the early stages of purification, copurifies with a high spin heme b protein seemingly not related to the supercomplex. The lower band copurifies with the supercomplex and is believed to represent an incompletely dissociated subcomplex containing cyt *cc*.

Fig. 8 shows SDS-gel analysis of pooled fractions at each step of another preparation. The image in the *left panel* shows the Coomassie-stained gel. In this preparation, the density gradient was run as the last step in order to avoid overloading the gradients with the large amount of protein present initially. The strong band at 50 kDa due to the supercomplex becomes visible after the DEAE-Sepharose chromatography (*lane 4*) and is slightly enriched by Q-Sepharose and Sepharose 6B chromatography. Finally, a major purification is accomplished by the density gradient. The *right panel* shows the same gel stained for covalent heme, before Coomassie staining. In addition to the expected band at 26 kDa due to cyt *cc*, two high molecular mass bands of ∼100 and 115 kDa are stained.

Neither of these heme-staining bands is apparent if the sample is heated to 95 °C in SDS denaturing buffer (not shown) before loading. The lower band is only seen in fractions containing supercomplex and probably represents a partially dissociated subcomplex containing cyt *cc*. The upper heme-staining band can be purified away from the supercomplex and is associated with heme B and a native spectrum consistent with a high spin heme. This band is about 120 kDa and coincides with a Coomassie-staining band that disappears on heat treatment as an ∼25 kDa band appears.

In these preparations, the detergent extract contained about 0.14 μmol of heme A, 0.35 μmol of heme B, and 0.08 μmol of heme C per g of starting protein. The value for heme C is not very accurate, because it is determined in the presence of a much greater amount of heme B, and the bands overlap. Still, there is significantly less heme C than heme A, and the two should be equal if all were present in fully assembled supercomplex containing heme A_2_B_2_C_2_ as determined below for the purified protein. (The amount of heme B is expected to be greater because other *b* cytochromes are present). Niebisch and Bott (15) have shown that in *C. glutamicum*, cytochrome *aa*_3_ is present in a mutant lacking the *qcr* operon. It may be that *qcr* is underexpressed in our transgenic strain, and excess oxidase accumulates as a result. A typical yield of purified supercomplex (monomer) was 9 nmol/g of starting protein, corresponding to ∼22% yield based on heme C or 13% based on heme A.

### Characterization

#### 

##### Size of the Supercomplex

Table 1 summarizes putative subunit composition of actinobacterial *bcc-aa*_3_ complexes. Ten subunits were associated with the *C. glutamicum* supercomplex (16). Seven of these, including the largest six, are also present in the *qcrCAB* operon of *M. tuberculosis* and *ctaC*, -*D*, -*E*, and -*F* of *M. smegmatis*. It is likely that the other three are present in the genome, but low sequence conservation in these small “picket fence” subunits (28) makes it difficult to identify them by database searching alone.

**TABLE 1:**
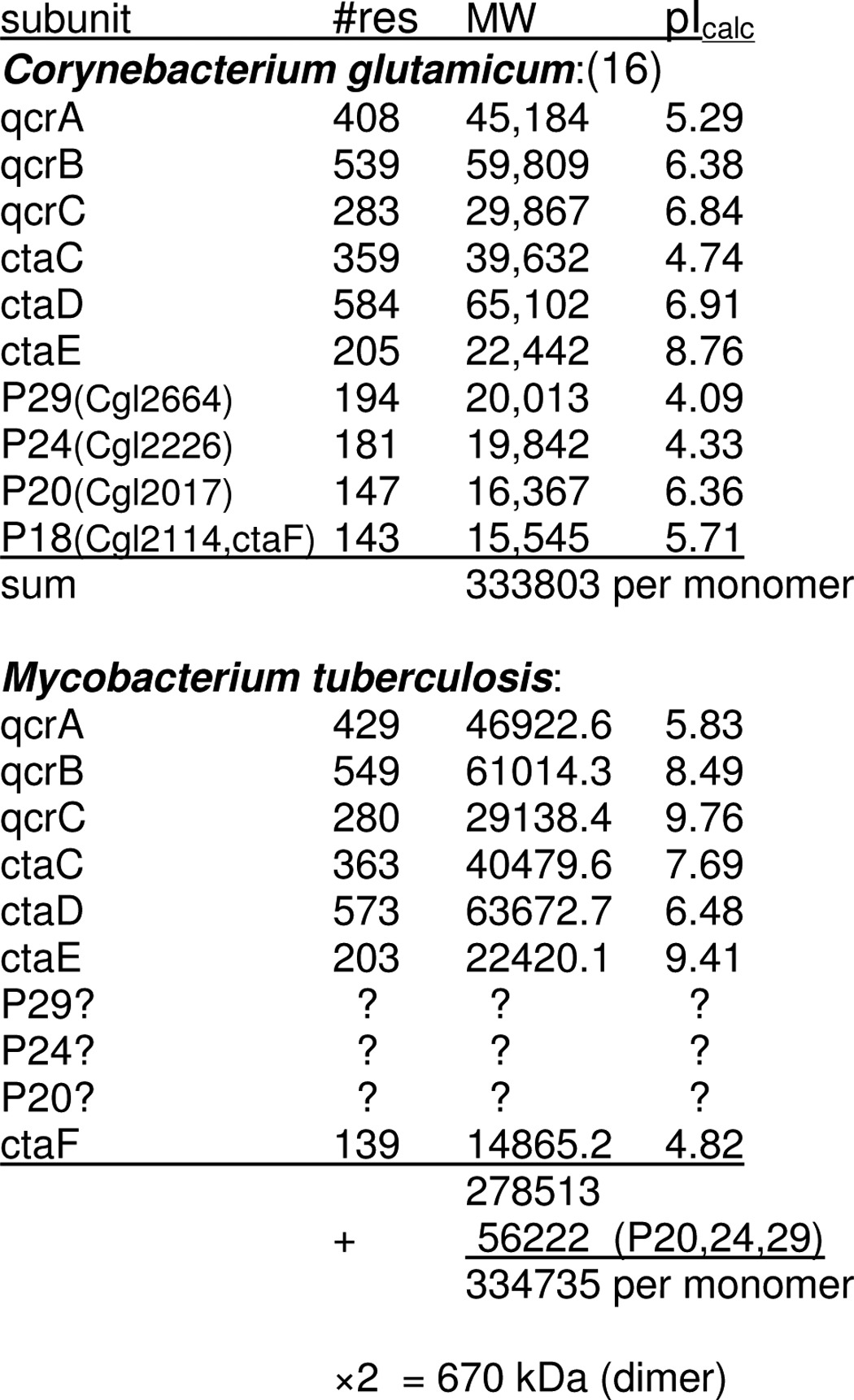
**Subunits of actinobacterial *bcc-acc*_3_ supercomplex**

The sum of the molecular mass of the seven known polypeptides (*M. tuberculosis qcrCAB* and *M. smegmatis ctaCDEF*) is 279 kDa (Table 1). Assuming that the homologues of *C. glutamicam* subunits P29, P24, and P20 are present and have the same molecular weights in *M. smegmatis*, they would add 56 kDa for a 335-kDa complex containing one copy of each subunit. Considering that all known *bc*_1_ structures are obligatorily dimeric due to crossover of the iron-sulfur protein extrinsic and membrane domains (29), together with the 1:1:1 heme ratio (determined below), the most likely arrangement is two of each subunit, with a molecular mass of 670 kDa. One step of the purification involves chromatography on a 200-ml Sepharose CL-6B column, from which it elutes at 0.57 of the salt elution volume. The column was not calibrated, but bovine *bc*_1_ (480 kDa) elutes from this column at 0.63 of the salt volume, so the supercomplex is significantly larger, possibly 670 kDa.

##### Subunit Composition

**FIGURE 9.**
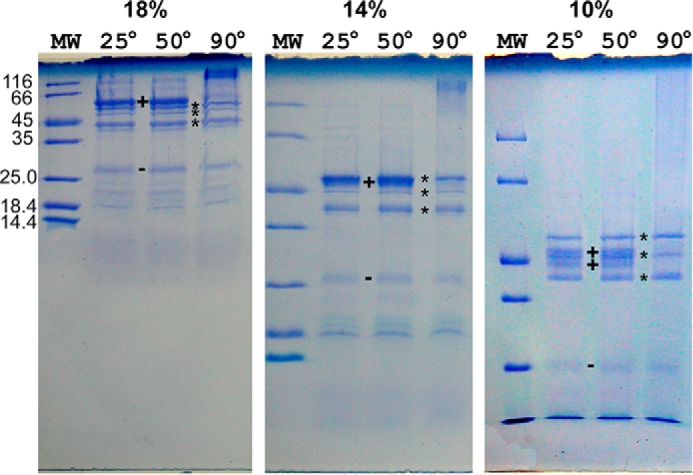
**Dependence of PAGE banding pattern of cyt *bcc-aa*_3_ on denaturation temperature and gel concentration.** The gels were made with different acrylamide concentrations (T+C) of 18, 15, and 10% (*left* to *right*). In each gel, the *first lane* is loaded with standards of the indicated molecular weight, and the other three are loaded with 13 pmol of cyt *bcc-aa*_3_ denatured for 15 min at room temperature, 50 °C, or 90 °C. Cyt *b* and perhaps Cox1 aggregate upon heating to 90 °C, leaving three sharp heat-stable bands (*) in the 45 kDa region. The band(s) that disappear on heating (+) have anomalous dependence on gel concentration, in that with changing gel concentration, their position changes relative to the heat-stable bands and molecular weight standards. Cytochrome *cc* (QcrC, 30 kDa) is identified by heme stain and labeled (−). Smaller subunits, visible in the 18 and 14% gels and failing to unstack in the 10% gel, may correspond to small proteins (P24, P20, and P18) identified in the *Corynebacterium* complex (16).

Fig. 9 shows SDS-PAGE analysis of purified supercomplex denatured for 15 min at different temperatures and run on gels of different acrylamide concentration. The 26 kDa band indicated by a *plus sign* stains for covalent heme and so must be cyt *cc*. Samples denatured at room temperature or 50 °C and run on a 14% gel show a very strong band at 50 kDa with a weak band just below it, and a moderate band at 40 kDa. When denatured at 90 °C, intensity of the 50 kDa band is decreased to that of the 40 kDa band, and staining material near the top of the gel indicates that the missing protein has aggregated. This is characteristic of large hydrophobic proteins, such as cyt *b* (30, 31) and Cox1.

Even when denatured at a temperature that does not lead to aggregation, large hydrophobic proteins, such as cyt *b* (30, 31) and Cox1 (32), from various organisms migrate anomalously on SDS gels, as indicated by a Ferguson plot. To test this and to find conditions under which subunits separate clearly for isolation and sequence determination, we ran the same sample on gels of 18, 14, and 10% acrylamide (T+C).

The protein(s) that disappeared on heating (Fig. 9, +) run anomalously with respect to gel concentration. On the 18% gel, it is above the heat-stable 50 kDa band, whereas on the 10% gel, it has moved down to the level of the weak middle band and seems to be splitting into two bands. If these indeed represent cyt *b* (61,014 kDa) and Cox1 (63,672 kDa), the bands remaining after 90 °C treatment must be *qcrA* (45,184 Da, the iron-sulfur protein) and *ctaC* (39,632 Da, Cox2), in which case the weak band in the middle may be an unknown component or contaminant.

The small subunits are not clearly defined by the gels of Fig. 9, which were loaded to optimize visualizing the large subunits, but there appear to be multiple subunits in the 19–20 kDa range. These may include *ctaE*, *ctaF*, and other small subunits.

Mass spectroscopic analysis of tryptic digests of the gel bands positively identified the 40 kDa band as *M. smegmatis* Cox2 (CtaC) and the weak band between 40 and 50 kDa as *M. tuberculosis* QcrA, the Rieske protein. These were from material separated on SDS gel after heating to 93 °C for 15 min. As in Fig. 9, this resulted in diminishing and sharpening the band at 50 kDa, together with the appearance of aggregated protein near the top of the gel. The sharp 50 kDa band gave positive identification (confidence interval 100%) of both the Rieske protein and Cox1 (CtaD). No positive identification at the 0.05 confidence level was obtained for the material at the top of the gel, but the highest score was for *M. smegmatis* Cox1 protein (CtaD). Cytochrome *b* was not identified (among the top five hits) in any of the bands, although we know from the spectrum that it was present in the sample applied.

An explanation consistent with these identifications is that the 50 kDa band contains Cox1 and cyt *b*, as in *Corynebacterium* (16), but also part of the Rieske protein. The Rieske protein runs as a doublet, due to proteolysis, partial denaturation, or post-translational modification, giving the intermediate band. The lower (40 kDa) band is Cox2. Upon heating, cyt *b* aggregates, runs near the top of the gel, and resists proteolysis and ionization. A small amount of Cox1 also aggregates and is detected near the top but at an insignificant level.

Weak bands at ∼34 and ∼26 kDa were also analyzed. Neither led to significant identification; however, we know from heme staining that the 26 kDa band is cyt *cc* (29,138 Da). Together with the spectral properties and the molecular mass from size exclusion (a little greater than 480 kDa), there can be little doubt that the product obtained is a supercomplex of cyt *bcc* and cytochrome *aa*_3_.

##### Spectral Characteristics, Heme Content, and Extinction Coefficients

One of purest fractions from a final glycerol density gradient was used to acquire standard spectra and subsequently denatured in alkaline pyridine solution to determine heme by the pyridine hemochrome method.

**FIGURE 10.**
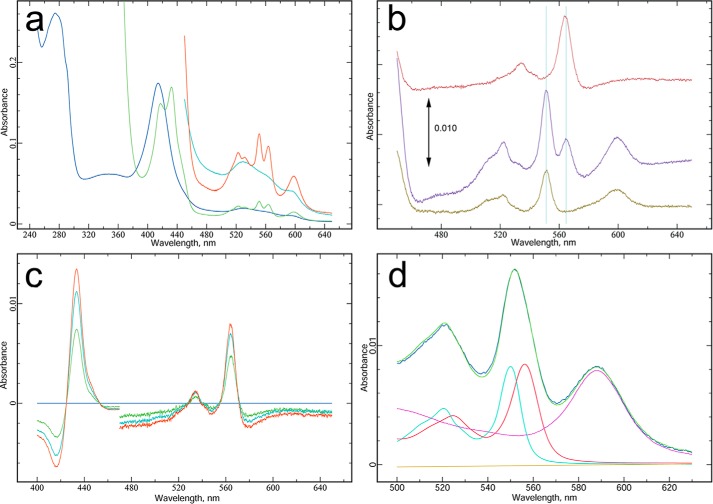
**Spectral properties of purified cytochrome *bcc-aa*_3_.** *a*, native spectra before (*blue* and *cyan*) and after (*green* and *red*) reduction with dithionite. Cytochrome *bcc-aa*_3_ was 25 μm, in 20 mm KMOPS 7.4, 100 mm NaCl, 0.5 mm EDTA, and 0.1 g/liter DDM. The *expanded cyan* and *red traces* are multiplied by 4. Spectra were taken with a Shimadzu UV-2450, bandpass 2 nm, scan speed fast. For *b* and *c*, another sample of cytochrome *bcc-aa*_3_ was first reduced with 0.17 mm ascorbate + 1.7 mm KCN and then with dithionite. Spectra were obtained at intervals. *b* (from *bottom* to *top*), change on adding ascorbate +KCN (*brown*), further immediate change on adding dithionite (*magenta*), and slow change after adding dithionite (*red*). *Vertical lines*, positions of the α peaks in the middle spectrum. In *c*, the first spectrum after dithionite (*gray horizontal line*) was subtracted from all to obtain intermediate steps during the slow reduction of cytochrome *b* in the subsequent (*green*, *cyan*, and *red*) spectra. *d*, cytochrome *bcc-aa*_3_ contains hemes A, B, and C in a 2:2:2 ratio. After recording the spectra of *a* with sodium dithionite added, an equal volume of 40% pyridine, 0.2 m NaOH (also treated with a small amount of dithionite) was added. The cuvette was stoppered and mixed well by repeated inversion. The *dark blue spectrum* was recorded from this mixture. The *green spectrum* is the best fit to this using the three absolute reduced pyridine hemochrome spectra and a linear baseline. The *cyan*, *red*, and *magenta* spectra indicate the amount of each used, respectively (0.253 μm heme C, 0.241 μm heme B, and 0.250 μm heme A), and *brown* is the baseline. The concentration based on a monomer containing two of each type of heme is obtained by dividing the sum of these by six. The value of 0.248 used for normalizing the spectra of *a* to obtain extinction coefficients was obtained by correcting this for the 2-fold dilution upon adding alkaline pyridine solution.

The native spectra of the untreated and dithionite-reduced supercomplex are shown in Fig. 10*a*. The oxidized form has maxima at 274.5, 414.5, and 529 nm, with minima at 312 and 371 nm. The ratio of the protein peak to Soret peak in the oxidized enzyme (*A*_274_/*A*_414_) was 1.50. The dithionite-reduced form has absorption maxima at 417 and 432.5 nm in the Soret region and 552.5, 564, and 599 nm for the α peaks. Notably, the peak position at 599 nm is significantly different from the peak for reduced bovine cytochrome oxidase around 605 nm. The peak at 417 nm suggests that the cytochrome is incompletely reduced, although this spectrum was acquired 33 min after adding dithionite.

Fig. 10*b* shows difference spectra obtained on treating another preparation with ascorbate/KCN and then dithionite. Cytochrome *cc* is incompletely reduced by ascorbate, which could be due to a low potential for one of the hemes or to failure of the cytochrome to reach equilibrium with ascorbate due to slow oxidation by a cyanide-insensitive pathway. The remaining *c*-type cytochrome is completely reduced, along with part of the *b* cytochrome, in the first spectrum after adding dithionite. The remainder is reduced slowly over the next 30 min, giving the difference spectrum in the upper trace.

Spectra recorded during that interval are shown in Fig. 10*c*, with the first spectrum after adding dithionite subtracted to show the slowly reduced component. This has an α peak at ∼564 nm and isosbestic points at 425, 452, 528, 540, 555, and 570 nm, suggesting that a single cytochrome is involved.

The spectrum in alkaline pyridine is shown in Fig. 10*d*, together with its decomposition into a linear combination of the three standard pyridine hemochromogen spectra. The least squares best fit was obtained with spectra corresponding to a ∼0.24 μm concentration of each of the three hemes. Equivalence of heme B and heme C is expected because the *bcc* complex contains two of each, and the near equivalence of heme A indicates that there is one oxidase per *bcc* complex rather than a single oxidase servicing both monomers of the dimeric *bcc* complex. Thus, we can express concentration of the supercomplex in terms of a monomer containing two of each type of heme. The concentration of the monomer in the alkaline pyridine solution of Fig. 10*d* is then 0.12 μm, and in Fig. 10*a*, before dilution with pyridine, it is 0.24 μm. This has been used to normalize the spectrum in Fig. 10*a* to obtain single and dual wavelength extinction coefficients for the supercomplex in the oxidized state. The extinction coefficient at the Soret peak (413 nm) was 726 mm^−1^, and the difference extinction coefficient at 413–371 nm was 485 mm^−1^.

Although extinction coefficients for the dithionite-treated enzyme could also be calculated, they would not be very meaningful in light of the incomplete and variable reduction of the cytochrome *b* obtained with dithionite. An exception would be the 599 nm peak due to oxidase, which should be minimally affected by a small amount of oxidized cyt *b* remaining. The extinction coefficient of the reduced form at 599–650 nm was 48. Accurate concentrations could be obtained from spectra of the reduced form by fitting the experimental spectrum with the absolute reduced spectrum (Fig. 10*a*) together with a difference spectrum for the slowly reducible cytochrome *b* (Fig. 10*c*).

To approximate the heme content on a protein basis, a sample of the pooled fractions from the same preparation was used for native oxidized spectrum and protein determination by the Lowry method. The ratio *A*_280_/*A*_415_ for this sample as 1.45, and *A*_415_ − *A*_371_ was 0.531, indicating a concentration of 1.11 μm. The protein concentration was 0.56 g/liter, giving an effective monomeric molecular mass of 478 kDa. The concentration dependence of the Lowry response did not match the BSA calibration curve very well. It is well known that proteins vary significantly in their response to the Lowry reaction, so this is not inconsistent with the molecular weight suggested by size exclusion chromatography or the sum of molecular weights of the subunits (Table 1).

### Conclusions

The hybrid supercomplex of *M. tuberculosis* cyt *bcc* and *M. smegmatis* cyt *aa*_3_ is stable during extraction and purification in the presence of dodecyl maltoside detergent. It is hoped that this purification procedure will potentiate functional studies of the complex as well as crystallographic studies of drug binding and thus contribute to the global fight against tuberculosis.

## Footnotes

8 The abbreviations used are:

IP: imidazo[1,2-*a*]pyridine
DDM: *n*-dodecyl β-d-maltoside
cyt: cytochrome
SU_IV_: subunit 4 of photosynthetic *b*_6_*f* complex
TMH: transmembrane helix.
